# Effect of Mo Oxides on the Phase Composition and Characteristics of Mo-10Re Pre-Alloyed Powders Co-Reduced with NH_4_ReO_4_

**DOI:** 10.3390/ma16175936

**Published:** 2023-08-30

**Authors:** Yi Zeng, Chaoping Liang, Yuanjun Sun, Na Wang, Xiangdong Ding, Jun Sun

**Affiliations:** 1 State Key Laboratory for Mechanical Behavior of Materials, School of Materials Science and Engineering, Xi’an Jiaotong University, Xi’an 710049, China; zengyiyihao1@163.com (Y.Z.);; 2 Jinduicheng Molybdenum Co., Ltd., Xi’an 710077, China; 3 Shaanxi Nonferrous Yulin New Materials Group Co., Ltd., Yulin 719099, China; 4 State Key Laboratory of Powder Metallurgy, Central South University, Changsha 410083, China

**Keywords:** co-reduction, Mo-10Re alloys, pre-alloyed powders, alloying mechanism

## Abstract

Mo-Re pre-alloyed powders are crucial raw materials in fabricating Mo-Re alloys, and their properties can significantly impact the properties of the resulting alloys. The powders are usually produced by the co-reduction of a mixture of Mo and Re oxides. However, it remains unclear if the overall characteristics of the produced Mo-Re powders rely on the different combinations of the Mo and Re oxide precursors. Therefore, in this work, a comparative study is conducted on the co-reduction processes of different Mo oxides together with NH_4_ReO_4_, along with its influence on the size distribution and phase composition of the resulting Mo-10Re pre-alloyed powders. The results show that MoO_3_ is more promising than MoO_2_ as a precursor material. The powders fabricated using MoO_3_, when compared to MoO_2_, have a much more uniform size distribution, with a primary particle size ranging from 0.5–4 μm. In addition, it is also beneficial to achieve atomic-scale homogeneous mixing with Mo and Re elements and the formation of a solely Mo(Re) solid solution if MoO_3_ is used as a precursor oxide. In contrast, such desirable features were not identified when using the MoO_2_ route. The reason for this discrepancy may relate to whether Mo-O-Re metallurgical bonding has formed during the co-reduction process.

## 1. Introduction

Refractory metal molybdenum possesses several characteristics, such as a high melting point, excellent mechanical properties at elevated temperatures, and impressive thermal conductivity, which make it highly suitable for applications in extremely harsh environments such as high-temperature, corrosive, and heavily irradiated conditions. Additionally, molybdenum demonstrates exceptional resistance to radiation-induced swelling and good compatibility with liquid alkali metals. Consequently, it emerges as a promising candidate for structural materials in advanced nuclear reactors, including the Space fast neutron reactor and fusion reactors [1,2,3,4,5]. However, the inherent brittleness at room temperature, as well as poor workability and welding performance, restrict the wide application of molybdenum. Nevertheless, when molybdenum is alloyed with rhenium (Re), it significantly enhances the ductility and tensile strength of the base metal due to the so-called “rhenium effect”. This improvement in mechanical properties persists even when the material is subjected to elevated temperatures that are higher than the recrystallization temperature [6,7,8]. Consequently, the former Soviet Union considered applying it to the preparation of cladding materials for the Bouk reactor [9]. In recent years, the Prometheus space reactor, the Idaho catalog gas-cooled reactor, and the RAPID-L fast reactor have adopted or are planning to adopt Mo-47.5Re as the cladding material and cooling circuit material for the core [10,11,12]. 

**Figure 1:**
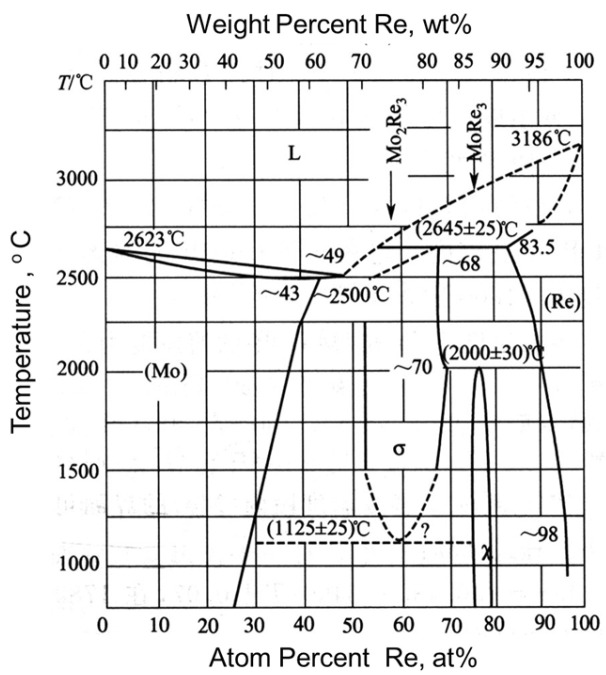
The Mo-Re phase diagram [16].

Mo-Re alloy is a solid-solution alloy that exhibits enhanced plasticity and workability due to the “rhenium effect.” Extensive research has been conducted to investigate the underlying mechanisms, summarized as follows: (1) The addition of rhenium to molybdenum results in the formation of an alloy that undergoes deformation not only through dislocations but also through twinning at room temperature. (2) The inclusion of rhenium induces a “solid-solution softening” effect, which reduces the hindrance to dislocation slip within the alloy. (3) Rhenium also increases the solid solubility of oxygen in the alloy and facilitates the formation of specific oxides. This overcomes the challenge of oxide segregation at the grain boundaries observed in pure Mo alloys, enhances grain boundary strength, and improves room temperature plasticity [13,14,15]. However, to fully utilize the merits of Mo-Re alloys, it is crucial to achieve a sufficient solid solution between rhenium and the matrix molybdenum. The phase diagram of the Mo-Re alloy (Figure 1) [16], illustrates that rhenium has a maximum solubility of 39 wt.% in molybdenum at room temperature. Above this solubility limit, the intermediate σ- and χ-phase phases form in the alloys, significantly reducing the mechanical properties of the material [17]. As a result, controlling the precipitation of these intermediate phases during material preparation, deformation processes, and material service becomes essential in fully harnessing the desirable merits of Mo-Re alloys [18,19]. 

Currently, Mo-Re alloys in the industry are prepared using powder metallurgy techniques. This involves the mixing of Mo powder and Re powder, followed by pressing, sintering, and deformation processes to obtain the final Mo-Re products. During these procedures, the alloying of Mo and Re elements primarily occurs through the thermal diffusion of powder particles during sintering. However, the density of molybdenum powder and rhenium powder differs significantly. Moreover, commercial molybdenum powder and rhenium powder often possess distinct particle size distributions and powder morphologies due to the variations in their reduction processes. Consequently, achieving perfect homogeneity through conventional physical mixing becomes a big challenge in the sintering process [20,21,22]. On the other hand, the micro-segregation of Re in the powder stage may preserve and make it difficult to homogenize during the subsequent alloying processes, such as sintering and heat treatment [22]. This, in turn, can lead to the formation of undesired σ and χ phases, ultimately deteriorating the material’s properties. 

In order to address the challenge of achieving a uniform distribution of Mo and Re elements in traditional mixing processes, researchers have explored alternative methods to improve the performance of Mo-Re alloys. One approach involves the preparation of pre-alloyed powders by co-reduction, which involves mixing the oxides or precursor materials of different components together and allowing them to undergo alloying during the reduction process. For example, Lan [23] and Cheetham [24] employed ammonium tungstate and ammonium molybdate solutions as their raw materials, mixing them in a liquid–liquid phase, which was then spray-dried and calcined to obtain a highly uniform mixture of MoO_3_-WO_3_ hybrid powder. This powder was then co-reduced to prepare a W-Mo pre-alloyed powder. Similar to W-Mo pre-alloyed powder, Małgorzata Osadnik [25] added liquid NH_4_ReO_4_ to Mo powders to improve the uniformity of the precursor mixture. A Re-coated Mo composite powder was obtained by co-reducing it at 900 °C. Liu [26] compared the sintering properties of Mo-3Re alloy powders prepared by adding an ammonium perrhenate solution and Re powder to Mo powder, respectively. After the co-reduction, they found that the use of an ammonium perrhenate solution is more conducive to improving the performance of a Mo-3Re alloy. It is well known that there are several types of oxide materials (such as MoO_3_, MoO_2_, Mo, ReO_2_, NH_4_ReO_4_, etc.) that can be utilized for the co-reduction of Mo and Re oxides. However, Currently, industry research has primarily focused on improving the uniformity of mixing between Mo and Re oxide precursors. It remains unclear if the overall characteristics of the produced Mo-Re powders depend on the different combinations of the Mo and Re oxide precursors. Consequently, challenges arise in selecting co-reducing oxide raw materials, determining suitable mixing methods, and regulating co-reduction process parameters. Therefore, in this work, MoO_3_ and MoO_2_ are solid–solid mixed with NH_4_ReO_4_ individually and subjected to hydrogen co-reduction at different temperatures. A comparative study is conducted on the co-reduction processes of different Mo oxides, MoO_2_, or MoO_3_ together with NH_4_ReO_4_ and their influence on the size distribution and phase composition of the resulting Mo-10Re pre-alloyed powders. The insights gained from this research into the alloying mechanism could also offer guidance for optimizing the co-reduction process of other pre-alloyed powders, such as Mo-W and Mo-Ni alloys.

## 2. Materials and Methods

### 2.1. Raw Materials

The MoO_3_, MoO_2_, and NH_4_ReO_4_ powders used in this work were all provided by Jinduicheng Molybdenum Industry Co., Ltd., Xi’an, China. MoO_3_ had an F.s.s.s particle size of 5.2 μm in an agglomerated state and a purity of ≥99.95%. MoO_2_ had an F.s.s.s particle size of 3.8 μm and a purity of ≥99.95%, which was prepared by the continuous boat reduction of the selected MoO_3_ in a four-tube furnace at a temperature gradient of 300 °C/350 °C/400 °C/450 °C/550 °C. The NH_4_ReO_4_ was sieved by −80 mesh, with a purity of ≥99.99%. The characteristics of the various raw materials are shown in Table 1.

### 2.2. Methods

**Figure 2:**
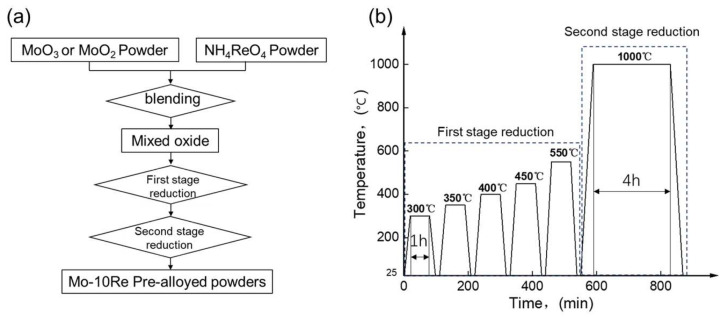
The flow chart and reduction cycle of the co-reduction process in this paper. (**a**) Flow chart; (**b**) Co-reduction cycle.

The selected MoO_3_ and MoO_2_ were ball milled with NH_4_ReO_4_ powders according to a metal Mo:Re ratio of 9:1 in those oxides. The milling time was 4 h (with ball) to a powder-to-weight ratio of 1:1, and the rolling speed was 30 r/min. To avoid contamination, the ball used for ball milling was a molybdenum ball with a diameter of 10 mm. The Mo-10Re pre-alloyed powder was prepared by using a two-stage reduction of the ball-milled mixtures in a single-tube reduction furnace. The first stage of reduction is the same as the MoO_2_ reduction process and is carried out according to a temperature gradient of 300 °C/350 °C/400 °C/450 °C/550 °C. For every 1 h of reduction at those temperatures, the material was taken out and characterized before the reduction at the next temperature. The hydrogen flow for first-stage reduction was 4 m^3^/min. After the first stage of reduction, the Mo-Re pre-alloyed powder was prepared by a subsequent second-stage reduction at 1000 °C for 4 h, and the hydrogen flow rate for that reduction was 6 m^3^/min. The flow chart and reduction cycle are shown in Figure 2.

### 2.3. Testing and Microstructure Characterization

The following instruments were used:

AutoChem II Automatic Chemisorption analyzer (TPR), Micromeritics, Chicago, USA; Miniflex600 X-ray diffractometer (XRD), Rigaku, Japan; Gemini 500 Scanning electron microscope (SEM), ZEISS, Oberkochen, Germany; FEI Titan3 Themis G2 scanning transmission electron microscope (TEM), FEI, Amsterdam, The Netherlands; Q600 Synchronous Thermal Analysis (DSC/TG), TA, Houston, TX, USA; WL-209 Fisher Sub-sieve Sizer, Dandong Aode Co., Ltd., Dandong, China; Single Tube Reduction Furnace, Xi’an Chenghang Co., Ltd., Xi’an, China.

Analytical Methods:

TPR test: The temperature range of the TPR test is 100–900 °C with a heating rate of 10 °C/min. The atmosphere of TPR is 90%Ar + 10%H_2_ mixed gas with a gas flow rate of 30.3 cm^3^/min. Degassing treatment was conducted at 100 °C for 15 min before the programmed temperature rise to eliminate the influence of moisture on the TPR tests; the weight of the sample test was 65.5 mg.

XRD detection: The light tube type was a Co target, ceramic X light tube. λ = 0.17889 nm, the scan range was 10–90 degrees, and the scanning speed was 5 degrees/min.

STD detection: The gas atmosphere was H_2_ with a flow rate of 40 mL/min during the test. The temperature range was 25 °C–500 °C, and the heating rate was 10 °C/min. The mass of the sample test was 31.5060 mg.

The parameters of SEM: The electron acceleration voltage was 20.0 KV, the working distance was 15 mm, and the magnification was 200–15,000 times.

The parameters of TEM: A high-angle annular dark field (HAADF) mode was employed to examine the powder morphology and elemental distribution. Prior to the microstructural examinations by STEM, the powders were first suspended in ethanol under ultrasonic vibration for 10~20 min, followed by transferring a few droplets (taken from the upper part of the suspension solution) to a 3 mm disc sample holder made of a copper grid and then drying the sample holder under an infrared lamp. When working, the electron acceleration voltage was 200 kV, and the magnification was 20,000~5,000,000 times.

## 3. Results

### 3.1. Powder Properties and Phase Composition

**Figure 3:**
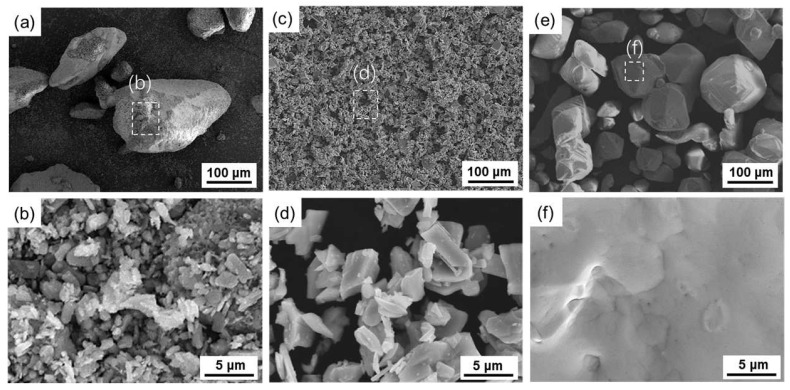
SEM characterization of raw materials used in this paper. (**a**,**b**) MoO_3_ powders; (**c**,**d**) MoO_2_ powders; (**e**,**f**) NH_4_ReO_4_ powders.

**Figure 4:**
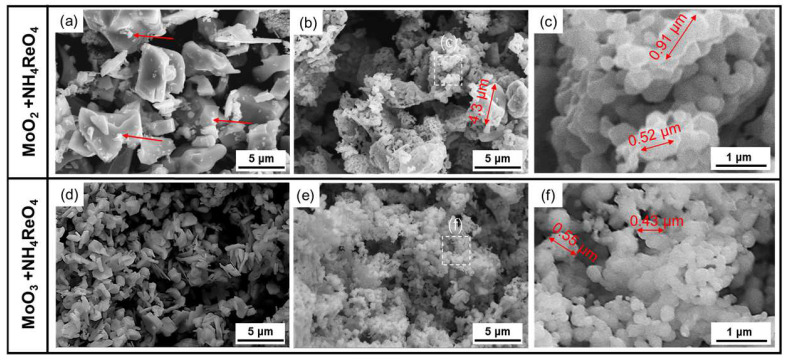
SEM characterization of mixed oxide powders after co-reduction. (**a**) MoO_2_ + NH_4_ReO_4_ mixture after the first stage of reduction at 550 °C; (**b**,**c**) MoO_2_ + NH_4_ReO_4_ mixture after the second stage of reduction at 1000 °C; (**d**) MoO_3_ + NH_4_ReO_4_ mixture after the first stage of reduction at 550 °C; (**e**,**f**) MoO_3_ + NH_4_ReO_4_ mixture after the second stage of reduction at 1000 °C.

Figure 4 shows the SEM characterization of the mixed oxide powders after co-reduction. It can be seen from Figure 4a that the mixture using MoO_2_ as the raw material after co-reduction at 550 °C does not show significant changes in terms of morphology when compared to the raw material (as shown in Figure 3d). The particle still shows a sheet morphology. Nevertheless, a large number of dispersed, small particles appear on the surface of the MoO_2_ particles (as indicated by the arrows in Figure 4a). This may be related to the hydrogen reduction of NH_4_ReO_4_. The morphology of the powder after the second reduction at 1000 °C is shown in Figure 4b,c. The powders consist of mainly primary particles, some of which have a size of less than 1 μm and exhibit certain agglomeration. Additionally, there are solid particles with diameters ranging from approximately 3 μm to 5 μm.

After the first stage of reduction, the particles of the mixture using MoO_3_ as the precursor are in the shape of long strips, and the particle size ranges from 1~3 μm (as shown in Figure 4d), which is only half that of the MoO_2_ raw material particles. It is noted that the MoO_2_ used in this test was prepared from MoO_3_ powder using the same first-stage reduction process. However, after being mixed with NH_4_ReO_4_ powder, the particle size of the powder obtained was significantly refined after the same reduction process, which shows that the addition of NH_4_ReO_4_ inhibited the growth of powder particles during the first-stage reduction process of MoO_3_. After the second stage of reduction at 1000 °C, the morphology of the powder can be seen in Figure 4e,f. When compared to the second stage of reduction of the MoO_2_ + NH_4_ReO_4_ mixture, the primary particles of the powder are finer and more uniform; most of the particles in the powders are below 1 μm.

**Figure 5:**
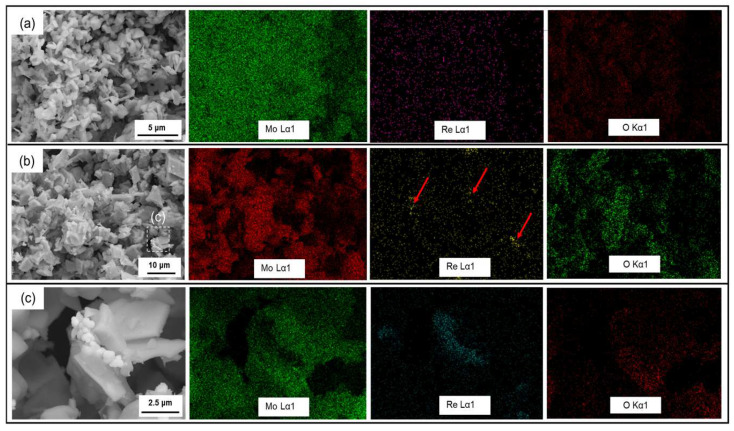
EDS mapping of powder after the first stage of reduction at 550 °C. (**a**) MoO_3_ + NH_4_ReO_4_ mixture after the first stage of reduction; (**b**,**c**) MoO_2_ + NH_4_ReO_4_ mixture after the first stage of reduction.

The EDS mapping of the powder after the first-stage reduction at 550 °C is shown in Figure 5. In the powder co-reduced by the MoO_3_+ NH_4_ReO_4_ mixture, the distribution of Re and Mo elements is relatively uniform, and there is no segregation of Re elements. On the contrary, when MoO_2_ was used, there was some Re element aggregation (shown as arrows in Figure 5b) in the powder produced by the same reduction process. The Re agglomeration area can be observed under high magnification in Figure 5c and the EDS mapping. It was found that Re was attached to the interlayer of MoO_2_, with particles of about 0.5~0.8 μm in size. The oxygen content in the Re enrichment area is relatively low. The above results show that under the same first-stage reduction process, using MoO_3_ as raw material is more conducive to the uniform distribution of Re in the powder than MoO_2_.

**Figure 6:**
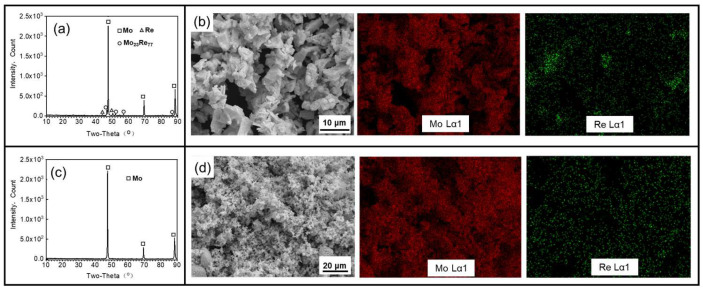
XRD patterns and EDS mapping of powders after the second stage of reduction at 1000 °C. (**a**,**b**) MoO_2_ + NH_4_ReO_4_ mixture after the second stage of reduction; (**c**,**d**) MoO_3_ + NH_4_ReO_4_ mixture after the second stage of reduction.

Figure 6 illustrates the XRD characterization and EDS mapping of the two powders after the second-stage reduction at 1000 °C. In Figure 6b,d, the distribution of Mo and Re elements indicates the presence of microsegregations in terms of the Re in the pre-alloyed powder prepared using MoO_2_ as the precursor. However, when MoO_3_ is used as the precursor, the distribution of Re appears more homogeneous. Figure 6c reveals that only molybdenum diffraction peaks are observed in the XRD pattern of the pre-alloyed powder prepared with MoO_3_ as the precursor. This suggests that Re exists primarily in the form of a Mo (Re) solid solution within this powder. Conversely, in the XRD pattern of the pre-alloyed powder using MoO_2_ as the precursor under the same reduction process, metal Re peaks and χ phase (Mo_23_Re_77_) diffraction peaks are observed in addition to the Mo peaks. This indicates that the Re is not completely dissolved in Mo, and the alloying of Mo and Re in the powder is insufficient. Although the presence of the Re-enriched phase in the pre-alloyed powder can be mitigated by employing higher sintering temperatures and longer sintering times in subsequent processes, it may still pose challenges to the overall sintering process and the performance of the alloy.

### 3.2. Alloying Mechanism and Phase Evolution

**Figure 7:**
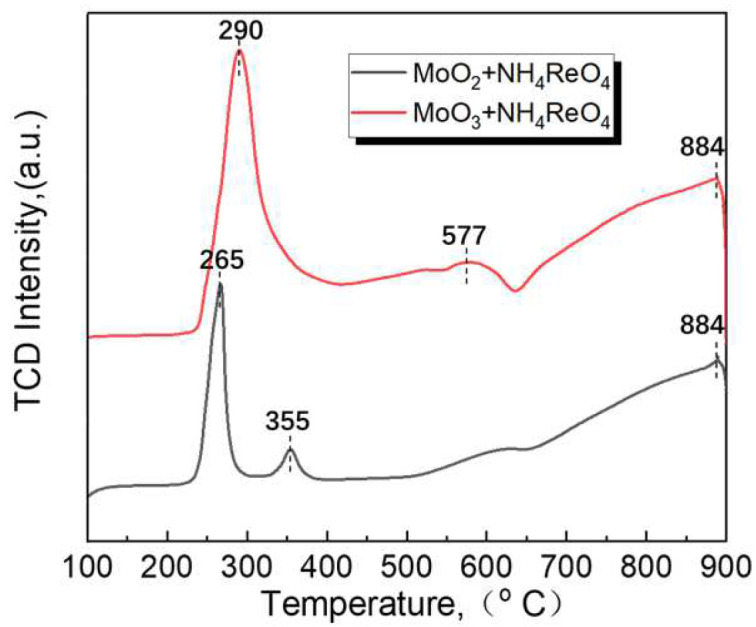
H_2_-TPR profiles of MoO_2_ + NH_4_ReO_4_ and MoO_3_ + NH_4_ReO_4_.

In order to compare the co-reduction process of the two mixed oxides used in this experiment, namely MoO_2_ and MoO_3_, temperature-programmed reduction (TPR) tests were conducted in a gas mixture of 90% Ar_2_ and 10% H_2_. The results are depicted in Figure 7. The horizontal axis represents the temperature, and the vertical axis represents the detected TCD signal, which is correlated with the H_2_ content in the gas after the reaction. Upon analyzing the H_2_-TPR curves, it is evident that the discrepancy between the two powders mainly lies at a temperature below 600 °C. When MoO_2_ was employed as the raw material, two reaction peaks appeared at 265 °C and 355 °C, respectively. On the other hand, when MoO_3_ was used, a broad reaction peak appeared at 290 °C. According to the reaction principles governing MoO_x_ hydrogen reduction [27,28,29], it is improbable for MoO_3_ and MoO_2_ to undergo hydrogen reaction conditions at temperatures below 360 °C. Therefore, the corresponding reaction peaks below 360 °C can be attributed to the reduction reaction of NH_4_ReO_4_ with H_2_. When MoO_2_ serves as the raw material, the two reaction peaks at 265 °C and 355 °C likely correspond to the reduction of NH_4_ReO_4_ to ReO_x_ and the subsequent reduction of ReO_x_ to Re metal, respectively. Conversely, when MoO_3_ is used, the reaction peak becomes wider, and the number of peaks decreases. This suggests that the hydrogen reduction process of NH_4_ReO_4_ may be influenced by MoO_3_, indicating a strong interaction between Mo-O-Re during the hydrogen reduction process. Furthermore, in both curves, the reaction peak appearing at 577 °C corresponds to the temperature at which MoO_3_ is reduced to MoO_2_, and the reaction peak at 884 °C corresponds to the temperature at which MoO_2_ is reduced to Mo by hydrogen.

**Figure 8:**
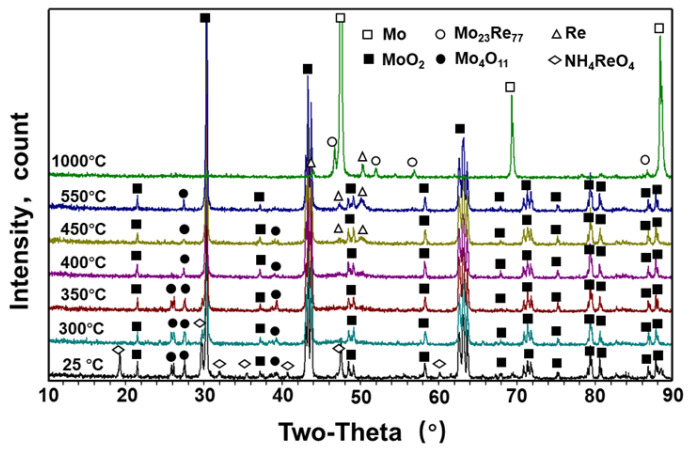
XRD patterns of powder reduced by MoO_2_ + NH_4_ReO_4_ mixture at 25–1000 °C.

XRD patterns of the powder reduced by the MoO_2_ + NH_4_ReO_4_ mixture at 25~1000 °C are shown in Figure 8. The NH_4_ReO_4_ + MoO_2_ mixed powder contains a small amount of Mo_4_O_11_, which may originate from the insufficient reduction of MoO_3_ in the MoO_2_ preparation process. This type of oxide will be gradually reduced to MoO_2_ again in the H_2_ atmosphere. From the temperature-dependent transformation of the phase containing Re in Figure 8, it is evident that, after reduction at 300 °C for 1 h, the diffraction peak of NH_4_ReO_4_ in the powder decreases. This indicates that NH_4_ReO_4_ begins to react with hydrogen gas at this temperature. After reduction at 350 °C for 1 h, the diffraction peak of NH_4_ReO_4_ disappeared, but the diffraction peak of the Re-related phase did not appear. This may be related to the partial overlap of the MoO_2_ peak or the incomplete crystallization of reduced Re-containing particles. As the reduction temperature further increases to 400 °C, the metal Re peak gradually appears. After reduction at 550 °C, the metal Re peak becomes clear in the diffraction peaks. After reduction at 1000 °C for 4 h, in addition to the metal Re diffraction peak, the Mo_23_Re_77_ diffraction peak also appears in the powder.

**Figure 9:**
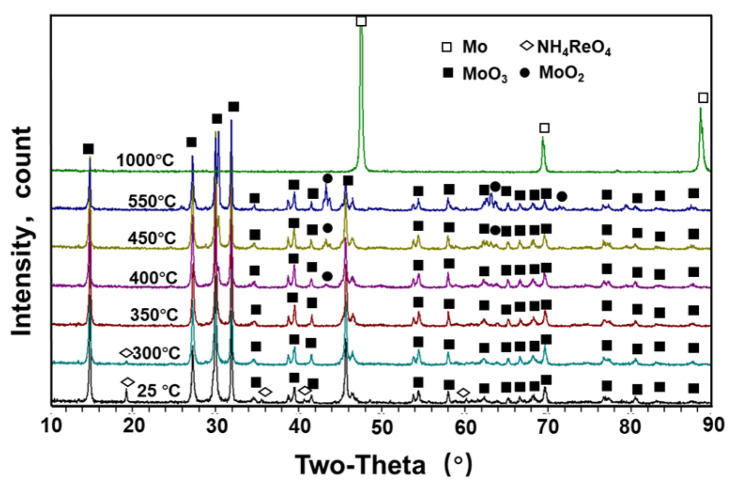
XRD patterns of powder reduced by MoO_3_ + NH_4_ReO_4_ mixture at 25–1000 °C.

The XRD diffraction of the MoO_3_ + NH_4_ReO_4_ mixture after co-reduction at different temperatures is shown in Figure 9. Similar to the results in Figure 8, the diffraction peak of NH_4_ReO_4_ gradually disappears between 300 °C and 350 °C. On the contrary, the disappearance of the diffraction peak of NH_4_ReO_4_ does not introduce a Re-related phase in the temperature range of 400–1000 °C. The reason for this phenomenon may be the superposition of the main peak of a Re-containing oxide with the diffraction peak of MoO_3_ or MoO_2_. Moreover, it may be related to the solid solution of ReOx in the lattice of MoOx, which was also confirmed by the following TEM characterization. From the temperature-dependent variation in the diffraction peak of the molybdenum-containing phase in Figure 9, a diffraction peak of MoO_2_ appears at 400 °C. After the first stage of reduction at 550 °C, the MoO_2_ content in the powder increases, but there is still a large amount of MoO_3_ in the powders. When using the same process to reduce MoO_3_, the powders were almost completely reduced to MoO_2_. This indicates that the addition of NH_4_ReO_4_ has a certain delaying effect on the reduction of MoO_3_. After reduction at 1000 °C, there is only a Mo peak in the powder XRD analysis, and MoO_X_ totally transforms into metallic Mo.

**Figure 10:**
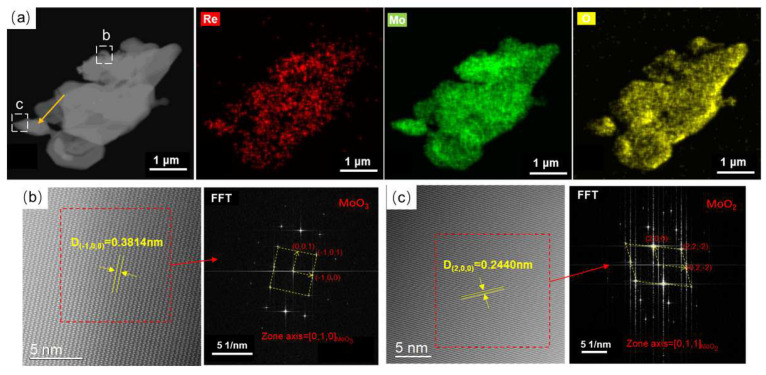
TEM analyses of MoO_3_ + NH_4_ReO_4_ powder reduction at 450 °C. (**a**) EDX mapping of the particle; (**b**) atomic image of selected area b; (**c**) atomic image of selected area c.

In order to determine the existing form of Re in the reduction process, spherical aberration TEM was used to characterize the MoO_3_ + NH_4_ReO_4_ mixed powder after hydrogen reduction at 450 °C, as shown in Figure 10. Figure 10a shows that the particle morphology of the powder monomer still retains the irregular particle morphology of MoO_3_. There are some finer particles about to be separated with a particle size less than 1 μm (as indicated by the arrow in Figure 10a). The EDS mapping results show that the distribution of Re in single particle is uniform, and there is no obvious aggregation in the micro-region. The atomic image characterization of the B and C regions are shown in Figure 10b,c, respectively. The FFT image and calibration results show that the phase structure in region C is similar to monoclinic-structured MoO_2_, whereas the same in region B looks like orthogonal-structured MoO_3_. This suggests that MoO_3_ is still the main component in the powder particles and that some MoO_2_ particles are reduced, which is consistent with the XRD results. Re is dissolved in the crystal lattice of MoO_3_ or MoO_2_, showing a uniform distribution.

**Figure 11:**
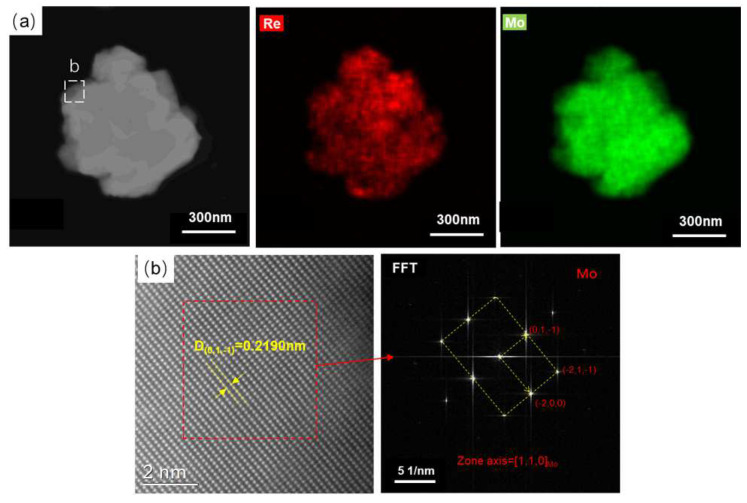
TEM analyses of MoO_3_ + NH_4_ReO_4_ powder reduction at 1000 °C. (**a**) EDX mapping of the particle; (**b**) atomic image of select area b.

After reduction at 1000 °C, the powder particle size observed by TEM was about 0.6 μm, as shown in Figure 11a. The EDS mapping results show that the Re is uniformly distributed in the molybdenum particles. HADFF atomic image observation was performed on the thin region of region b, as shown in Figure 11b, and FFT conversion and phase calibration were performed on it, which is close to the BCC-structured Mo phase. Therefore, it can be further confirmed that Re exists in the Mo matrix as a solid solution after reduction at 1000 °C, which is consistent with the XRD results.

**Figure 12:**
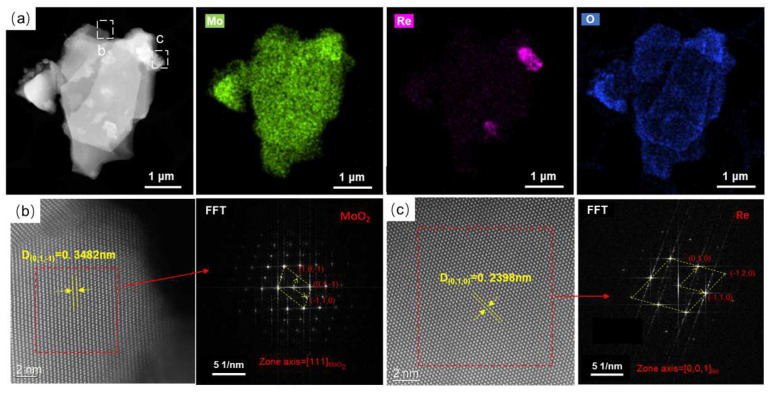
TEM analyses of MoO_2_ + NH_4_ReO_4_ powder reduction at 450 °C. (**a**) EDX mapping of the particle; (**b**) atomic image of select area b; (**c**) atomic image of select area c.

The TEM characteristics of the MoO_2_ + NH_4_ReO_4_ mixed powder after hydrogen reduction at 450 °C are shown in Figure 12. The results show that the powder monomer particles presented as layered shapes, with a particle size of about 4 μm. Unlike the results in Figure 10a, the Re is not uniformly distributed within the powder particles but is, rather, segregated on the surface of the particles. HADFF atomic imaging was used to characterize area b and area c in terms of the particles, and the results are shown in Figure 12b,c, respectively. After FFT conversion and calibration, it was found that the phase in the b region of Figure 12b is like monoclinic-structured MoO_2_, whereas the phase in the c region is similar to HCP-structured Re. This result implies that after the reduction at 450 °C, the NH_4_ReO_4_ in the powder was reduced to Re. The reduced Re is concentrated on the surface of the MoO_2_ particles.

**Figure 13:**
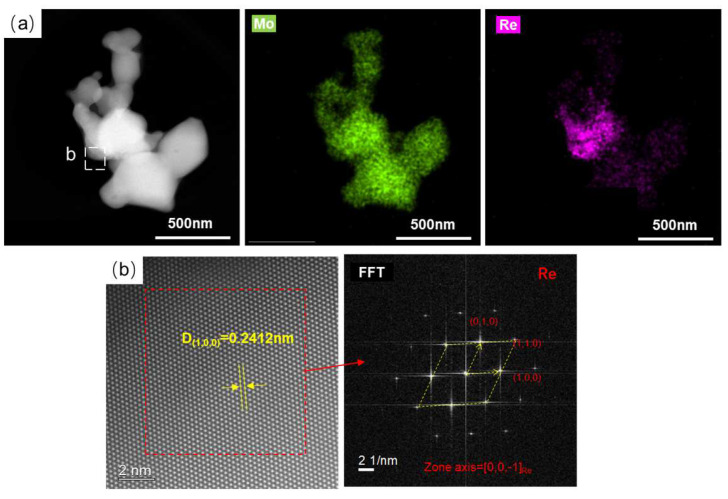
TEM analyses of MoO_2_ + NH_4_ReO_4_ powder reduction at 1000 °C. (**a**) EDX mapping of the particle; (**b**) atomic image of select area b.

The TEM characterization of the mixed powder MoO_2_ + NH_4_ReO_4_ after reduction at 1000 °C is shown in Figure 13. The observed powder particles are nearly spherical and exhibit some agglomeration. The EDX mapping shows that there are scattered intercalated Re particles in the agglomerated molybdenum particles. The atomic phase captured at the Re aggregation area is shown in Figure 13b. After FFT conversion and calibration, it was found that the Re in the b region is close to the HCP structure. This indicates that the aggregation phenomenon of Re after the first-stage reduction at 450 °C was inherited into the second-stage reduction at 1000 °C. In this powder, the particle size of Re agglomeration is around 200 nm, and it is connected to the surrounding Mo particles. The further away from the area of the Re aggregation particles, the higher the Mo content and the lower the Re content. Consequently, it is possible for Re to exist in the form of aggregated elemental Re, as well as (ReMo_x_) intermediate phases or Mo (Re) solid solutions at the interfaces between the Mo and Re particles. This explains why the XRD analysis revealed the presence of various phases, such as Re and Re_23_Mo_77_, within the powder, indicating the complex nature of Re distribution within the alloy.

## 4. Discussion

In the industrial production process, NH_4_ReO_4_, MoO_3_, and MoO_2_ are commonly employed as raw materials for the reduction of metal Re and Mo powder, respectively. Each of these materials follows their own reaction rules during reduction in a hydrogen atmosphere.

The reduction of NH_4_ReO_4_ typically undergoes a series of reactions, as per (1) to (6):

NH_4_ReO_4_(s) = HReO_4_(s) + NH_3_(g) (1)

2HReO_4_(s) = Re_2_O_7_(s) + H_2_O(g) (2)

Re_2_O_7_(s) + H_2_(g) = 2ReO_3_(s) + H_2_O(g) (3)

ReO_3_(s) + H_2_(g) = ReO_2_(s) + H_2_O(g) (4)

Re_2_O_7_ + 3H_2_(g) = 2ReO_2_(s) + 3H_2_O(g) (5)

ReO_2_(s) + 2H_2_(g) = Re(s) + 2H_2_O(g) (6)

The overall reaction formula is

2NH_4_ReO_4_(s) + 7H_2_(s) = 2Re(s) + 8H_2_O(g) + 2NH_3_(g) (7)

It is worth noting that the specific reaction sequence and temperatures may vary depending on factors such as oxygen partial pressure, hydrogen dew point, and the particle size of NH_4_ReO_4_ during the reaction process [30,31].

**Figure 14:**
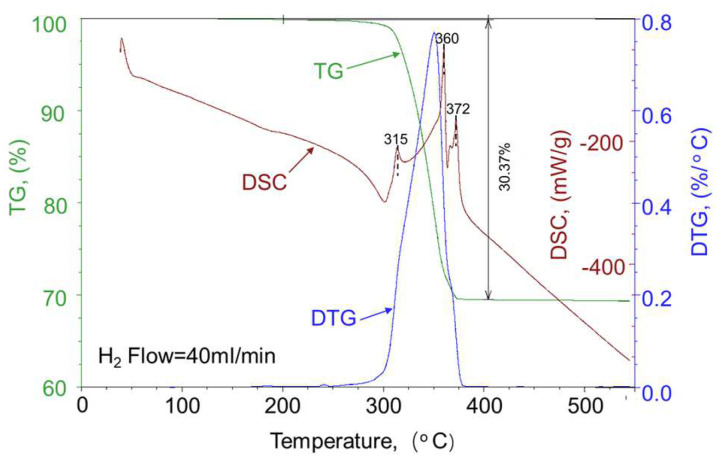
TG and DSC thermogram of NH_4_ReO_4_ used in this experiment.

Figure 14 displays the DSC-TG curve of the NH_4_ReO_4_ used in this experiment, with a hydrogen flow rate of 40 mL/min and a heating rate of 10 °C/min. The TG and DTG curves in the figure reveal that the reduction temperature range of NH_4_ReO_4_ mainly falls between 250 °C and 375 °C, with a peak reaction temperature at approximately 350 °C. The remaining weight after the reaction is 69.63%, which is in line with the Re content of NH_4_ReO_4_ (69.3 wt.%). This indicates that NH_4_ReO_4_ can be fully reduced to Re prior to 375 °C. Notably, the DSC curve exhibits exothermic peaks at 315 °C, 360 °C, and 372 °C, corresponding to the reactions outlined in Formulas (1) to (6). According to a literature report, oxides like Re_2_O_7_ and ReO_3_ have a high evaporation pressure (around 300 °C), making them prone to form evaporated substances within the powder [32]. The volatile nature of these process oxides, combined with the generation of H_2_O and NH_3_ during the reaction, leads to the hydrogen reduction process of NH_4_ReO_4_ following a gas phase migration model [31]. This gas phase migration model is preferable for achieving the homogenization of Re in Mo during the reduction process.

When MoO_3_ is reduced in a hydrogen atmosphere, it usually undergoes two stages of reaction. The first stage is MoO_3_ reacting with H_2_ to generate MoO_2_ at 450~550 °C, and the second stage is the MoO_3_ reaction with H_2_ to generate Mo at 850~900 °C [33]. The chemical vapor transport mechanism (CVT) has been proven to be the dominant mechanism in the first stage of reduction [34]. In this mechanism, MoO_3_ will form a vapor transition phase, mainly composed of Mo-O-H with H_2_O vapor in the atmosphere, which will evaporate and then deposit on the MoO_2_ core. However, the second stage of reduction has two mechanisms working together, i.e., the pseudomorphic transformation mechanism at a low dew point and chemical vapor migration at a high dew point. These two mechanisms have been confirmed by many researchers [33,35,36,37].

When NH_4_ReO_4_ and MoOx are mixed together, if they can react with H_2_ gas at the same temperature, the complex equilibrium of Mo-O-Re-H will form during the reaction process. In addition, MoOx and ReOx follow the chemical vapor phase transport mechanism in the hydrogen reaction process, which may lead to the co-nucleation of Mo and Re particles in the co-reduction process. These mechanisms will shorten the diffusion path of Mo and Re and lead to the alloying of them in the powder stage. However, the reaction temperature of NH_4_ReO_4_ in an H_2_ atmosphere is 250~400 °C, whereas that of MoO_3_ is 450~550 °C, and MoO_2_ is much higher. It seems that there is no common reaction temperature range between Re and Mo oxides.

The results of this experiment show that when MoO_2_ is used as raw material in the co-reduction process, the first stage of reduction is dominated by the reduction of NH_4_ReO_4_ to Re through a series of reactions. Although the NH_4_ReO_4_ particles are above 50 μm, the fine particle size of Re (<1 μm) is reduced, nucleated, and grows on the surface of the MoO_2_ particles through gas phase transport in the reduction process. This gas phase migration can improve the distribution of Re in the powder. Even so, after the completion of the first stage reaction, no obvious metallurgical bonding between Re and Mo particles can be detected in by TEM and XRD. During the second-stage reduction process of the powder at 850~950 °C, the oxygen in MoO_2_ will be reduced by hydrogen, and the area contacted by the MoO_2_ and Re particles will interdiffuse and form solid solution particles. However, some of the larger Re particles are not completely diffused into Mo under this reduction process, leading to the Re phase and Mo_23_re_77_ phase appearing in the powder. The alloying of Mo and Re in the powder is not perfect.

When MoO_3_ is used as raw material, NH_4_ReO_4_ and MoO_3_ also do not have a common co-reduction reaction temperature. It seems that NH_4_ReO_4_ and MoO_3_ will be carried out alternately according to their reaction temperature with hydrogen. However, from the comparative analysis results of TPR, it was found that the hydrogen reduction process of NH_4_ReO_4_ is obviously affected by MoO_3_ particles (as shown in Figure 7). The TEM and XRD analysis of the reduction products at 450 °C shows that there is a solid solution of ReO_x_ in the MoO_3_ particles. This means that the ReO_X_ particles reduced from NH_4_ReO_4_ will dissolve into the lattice of MoO_3_ at this temperature, and an evenly distribution of Mo and Re atoms is obtained. The reason for the solid solution may be related to the high diffusion coefficient of Re in MoO_3_, or it might attributed to the fact that Re can capture the O atom of MoO_3_ and form Mo-O-Re oxides. The specific reasons need to be further studied. With a further increase in the reduction temperature, the oxygen element in MoO_3_ is gradually reduced by H_2_ to MoO_2_ and Mo particles. The gas phase transport mechanism involved in this process will further make the distribution of Mo and Re elements more uniform. There is only a single Mo (Re) solid solution phase in the powder after reduction at 1000 °C, as confirmed by Figure 6b and Figure 11.

The experimental findings presented above highlight the significance of the solid solution between Re and MoO_3_ in the alloying process of the Mo-Re-O system after reduction. When employing oxide co-reduction for the preparation of Mo-Re pre-alloy powder, aside from ensuring the uniform mixing of the oxides, maximizing the utilization of the interaction between Mo-O-Re emerges as another crucial factor for obtaining a highly uniform pre-alloy powder. Therefore, in addition to focusing on optimizing oxide mix uniformity, it is highly recommended to explore and harness the potential interaction between Mo, O, and Re.

## 5. Conclusions

This paper presents a comparative study of the powder characteristics and phase composition of Mo-10Re pre-alloy powder co-reduced using MoO_3_ or MoO_2_ with NH_4_ReO_4_. The main conclusions of the study are as follows:

(1) When MoO_3_ is used as the raw material for co-reduction, the resulting pre-alloy powder exhibits a uniform size distribution with a primary particle size of less than 1 μm and consists solely of the Mo (Re) solid solution phase. In contrast, when MoO_2_ is utilized as the raw material, it yields pre-alloy powders with an uneven size distribution that ranges from 0.5~4 μm and contains undesirable Re and Mo_23_Re_77_ phases in addition to the target Mo (Re) solid solution phase.

(2) During the reduction process, NH_4_ReO_4_ was reduced to metallic Re at 300–450 °C and attached to the surface of MoO_2_ particles, whereas MoO_3_ adsorbed the reduced ReO_X_ and formed Mo-O-Re metallurgical bonding, which led to more homogeneous elemental mixing in the resulting Mo-10Re powders.

Based on these findings, MoO_3_ is considered a more promising oxide precursor than MoO_2_ for producing Mo-Re pre-alloyed powders. Furthermore, the insights gained from this work suggest that the metallurgical bonding of oxides during the co-reduction process can enhance the alloying effect of pre-alloyed powders.

## Figures and Tables

**Table 1:** Characteristics of the raw materials used in this study.

Powder	Purity	F.s.s.s Size, μm	Bulk Density, g/cm^3^
MoO_3_	≥99.95%	5.2	1.42
MoO_2_	≥99.95%	3.8	0.85
NH_4_ReO_4_	≥99.99%	≥50	1.12

The particle size of NH_4_ReO_4_ powder is too large to be measured by a Fisher sub-sieve sizer.

## Data Availability

Data are contained within the article.
